# Salivary Biomarker Levels and Oral Health Status of Children with Cerebral Palsy and Their Healthy Siblings: A Comparative Study

**DOI:** 10.5041/RMMJ.10437

**Published:** 2021-04-29

**Authors:** Palanichamy Anjugam, Veerabadhran Mahesh Mathian, Murugesan Gawthaman, Selvaraj Vinod, Easwaramurthy Yamuna Devi

**Affiliations:** Department of Pedodontics and Preventive Dentistry, Vivekanada Dental College for Women, Elayampalayam, Tiruchengode–TK Namakkal District, Tamil Nadu, India

**Keywords:** Cerebral palsy, dental caries, salivary biomarkers

## Abstract

**Background:**

The dental needs of cerebral palsy children are an area of study much in need of attention. The neglect of this aspect should be rectified, and simpler diagnostic methodologies should be established and used to serve this purpose.

**Aim:**

This study aimed to determine oral health status and salivary biomarkers (salivary flow rate, pH, buffering capacity) among children with cerebral palsy (CP), to compare their data with that of their healthy siblings, and to evaluate the relationship between salivary biomarkers and dental caries.

**Methods:**

A total of 30 CP children (study group) and 30 normal healthy siblings (controls) were selected between the ages of 5 and 12 years. Salivary biomarkers were assessed, and oral health status was examined.

**Statistical Analysis:**

Chi-square test was used for comparison of oral health status. Unpaired *t* test was used to compare caries indexes (decay/filled teeth–primary dentition [dft] and decay/missing/filled teeth–permanent dentition [DMFT]) and salivary biomarkers between the groups. Pearson correlation was used to find the correlation between salivary biomarkers and caries.

**Results:**

The dft scores were significantly higher in the study group (*P*<0.05). The pH values and salivary flow rates were significantly lower in the study group (*P*<0.05 and *P*<0.001, respectively). There was a significant correlation between DMFT scores and salivary flow rate in the study group (*P*<0.05).

**Conclusion:**

Low pH and low salivary flow rate might be risk factors for dental caries in CP populations; moreover, the significant correlation between DMFT score and salivary flow rate suggests that salivary flow rate could be used as a screening tool for assessing at-risk subjects in such populations.

## INTRODUCTION

Cerebral palsy (CP) was first described by Sir William John Little in 1862.^1^ The condition is described by Dr Winthrop Phelps as the disorder of movement and posture resulting from permanent static defect or lesion of the brain affecting mostly preterm infants.^2^ It results in severe dysfunctions and sensory and cognitive impairment. This subgroup of children needs full attention and extra care to perform their daily activities. Furthermore, studies have reported that special facilities and care are needed for CP children for everything from simple dental procedures to those performed under general anesthesia. Hence, a complete screening should be a part of their initial dental assessment. As self-care is challenging for these children, prevention of medical conditions and early treatment should be aimed at. However, only sparse data are available in the literature regarding the dental health of CP children and the use of easy diagnostic methods for caries (such as less invasive salivary sample).

This study sought to determine if there was a correlation between the occurrence and severity of dental caries and salivary flow rate, pH, and buffering capacity of the respective children. Specifically, we sought:

1. To determine the oral health status among CP children in comparison with their healthy siblings.
2. To determine the salivary biomarkers of CP children in comparison with their healthy siblings.
3. To evaluate if any relationship exists between the salivary biomarkers found in CP children and the presence of dental caries.

## MATERIALS AND METHODS

This study was approved by the institutional research ethics committee of Vivekananda Dental College for Women. Parents were informed about the purpose of the present study, and written informed consents for the participation of each CP child and their healthy siblings were obtained from their parents.

This study was performed with CP children who had received physical rehabilitation in the Christian Fellowship Hospital, Oddanchatram, Tamil Nadu. Thirty non-institutionalized children diagnosed with CP between the ages of 5 and 12 years as verified by their medical records (study group) and their 30 healthy siblings (control group), within the same age group, were included in this study. The healthy siblings were considered to be an ideal control group since there was a natural match between them and the study group for confounding factors affecting dental caries such as diet (nature, type, form, method of preparation), socioeconomic status, and parental knowledge of the children’s oral health. Exclusion criteria were children of parents who did not give consent for participation and those children who were unable to cooperate in the study. Two families were unable to participate on the day of sample collection and were considered to be drop-outs. Hence, a total of 56 children completed the study.

All examinations were performed at the hospital by a single examiner using a mouth mirror, explorer, a World Health Organization (WHO) periodontal probe with proper illumination, and universal infection control procedures. Oral health status was assessed using WHO oral assessment form for children 2013, and oral hygiene was assessed using the Simplified Oral Hygiene Index (OHI-S).^3^

For unstimulated whole saliva^4^ sample collection, the children fasted from food for at least 12 hours and from drinking water one hour before sample collection; samples were collected between 9 am and 12 noon. The passive drool method was used for sample collection in cooperative CP children. During collection, the children were asked to sit in a relaxed coachman position and asked to expectorate into clean, dry, sterilized glass tubes. The suction method was used to collect samples from the most severely challenged CP children.^4^,^5^ Control group samples were collected in a manner similar to that of the CP children. The samples were delivered to the Department of Pharmaceutics of Vivekananda College of Pharmacy for calculation of the pH, quantity, flow rate, and buffering capacity.

Quantity was calculated as the total amount collected in the graduated test tube at the end of 5 minutes; flow rate was calculated by measuring the quantity of unstimulated saliva in mL/min.^6^

Standard pH indicator strips were used, and the characteristic color change with reference to the standard pH strip was noted.^5^

Buffering capacity was measured using Ericsson’s method: 1 mL of a saliva sample was mixed with 3 mL of hydrochloric acid. One drop of 2-octanol was added to prevent foaming, and then mixed for 20 minutes to remove carbon dioxide. The final pH was evaluated electrometrically.^7^

### Statistical Analysis

Chi-square test was used for comparison of the oral hygiene index, gingival bleeding, dental trauma, mucosal lesions, dental erosion, and enamel fluorosis. Unpaired *t* test was used to compare the mean score values of the caries index and the salivary biomarkers between the groups. Pearson correlation was used to find the correlation between salivary biomarkers and caries. The significance level was established at *P*<0.05.

## RESULTS

A total of 28 CP children aged between 5 and 12 years and their 28 normal healthy siblings were examined. The results obtained were as follows.

**Figure 1:**
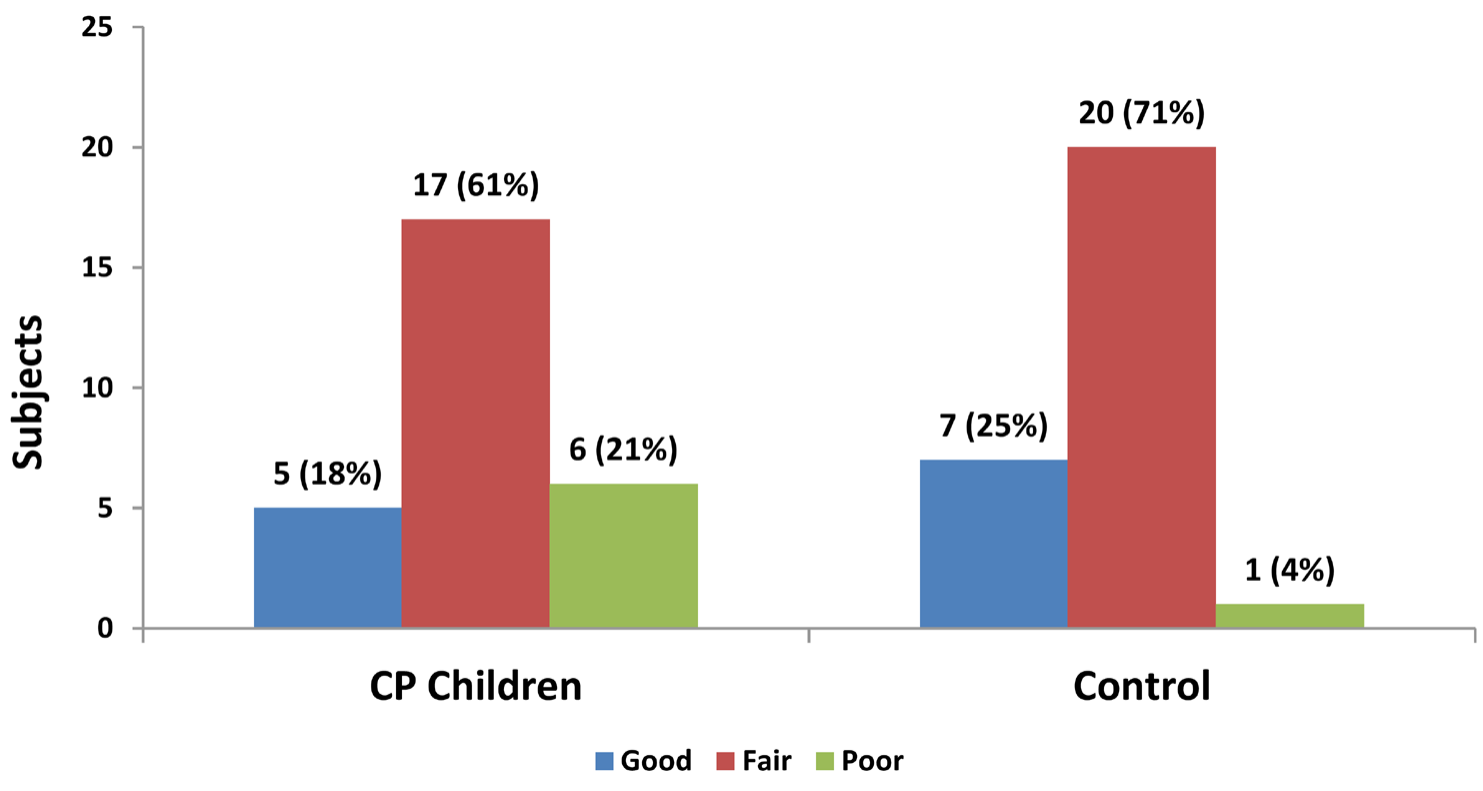
Comparison of Simplified Oral Hygiene Index (OHI-S) in CP Children and Controls.

Figure 1 shows the grading of oral hygiene. Differences observed between the CP children and the control group for poor oral hygiene were not significant (*P*>0.05).

Table 1 presents dental signs and symptoms found in the study group versus controls. There was no significant difference between the two groups.

**Table 1:** Presence of Dental Signs and Symptoms in CP Children versus Controls.

Parameter	CP Children *n* (%)	Control *n* (%)	*P* Value
Dental Trauma	6 (21)	2 (7)	>0.05
Gingival Bleeding	18 (64)	19 (75)	>0.05
Mucosal Lesions	7 (25)	2 (7)	>0.05
Dental Erosion	7 (25)	4 (14)	>0.05
Enamel Fluorosis	5 (18)	7 (25)	>0.05

Table 2 presents differences in dental caries status (dft [decay/filled teeth–primary dentition], DMFT [decay/missing/filled teeth–permanent dentition) and salivary biomarkers (pH, buffering capacity, saliva flow rate) between the study and control groups. Significant differences were noted for the dft, pH, and salivary flow rate.

**Table 2:** Comparison of Dental Caries Status and Salivary Biomarkers between CP Children and Controls.

Variable		Group	*n*	Mean	SD	*t*	*P*
Dental Caries	dft	Control	28	1.71	1.84	2.50	0.016*
		CP Children	28	3.61	3.56		

	DMFT	Control	28	1.25	1.32	0.22	0.828
		CP Children	28	1.18	1.12		

Salivary Biomarkers	pH	Control	28	5.68	1.59	2.48	0.016*
		CP Children	28	4.61	1.64		

	Buffering Capacity	Control	28	1.70	0.41	0.29	0.770
		CP Children	28	1.67	0.38		

	Salivary Flow Rate	Control	28	5.14	1.84	3.76	0.001**
		CP Children	28	3.25	1.93		

The dft score range is 0–18. The lower the score, the less the decay/number of filled teeth in primary dentition; the greater the score, the more the decay/number of filled teeth in primary dentition. The DMFT score range is 0–5. The lower the score, the less the decay/number of missing/filled teeth in permanent dentition; the higher the score, the more the decay/number of missing/filled teeth in permanent dentition.

* Significant at 0.05;

** Significant at 0.01

dft, decay/filled teeth–primary dentition; DMFT, decay/missing/filled teeth–permanent dentition.

Furthermore, a significant two-tailed correlation was seen only between salivary flow rate and dental caries in CP children (Table 3).

**Table 3:** Correlation of Dental Caries and Salivary Biomarkers in CP Children.

Variable		Correlation	dft	DMFT	pH	Buffering Capacity	Saliva Flow Rate
Dental Caries	dft	Pearson Correlation	1	0.018	−0.217	0.050	−0.009
		Sig. (2-tailed)	-	0.927	0.266	0.799	0.964
		*n*	28	28	28	28	28

	DMFT	Pearson Correlation	0.018	1	0.341	−0.036	0.461*
		Sig. (2-tailed)	0.927	-	0.076	0.855	0.014
		N	28	28	28	28	28

Salivary Biomarkers	pH	Pearson Correlation	−0.217	0.341	1	0.126	0.356
		Sig. (2-tailed)	0.266	0.076	-	0.525	0.063
		*n*	28	28	28	28	28

	Buffering Capacity	Pearson Correlation	0.050	−0.036	0.126	1	0.193
		Sig. (2-tailed)	0.799	0.855	0.525	-	0.326
		*n*	28	28	28	28	28

	Salivary Flow Rate	Pearson Correlation	−0.009	0.461*	0.356	0.193	1
		Sig. (2-tailed)	0.964	0.014	0.063	0.326	-
		*n*	28	28	28	28	28

dft score range, 0–18; DMFT score range, 0–5.

* Correlation is significant at the 0.05 level (two-tailed).

dft, decay/filled teeth–primary dentition; DMFT, decay/missing/filled teeth–permanent dentition.

## DISCUSSION

Jan et al. emphasized that for spastic CP children, treatment modalities should be mainly aimed at improving mobility, reducing or preventing contractures, improving positioning, and focusing on hygiene.^1^ However, this study found no significant differences in terms of oral hygiene status, gingival bleeding, dental erosion, dental trauma, mucosal lesion, or enamel fluorosis between the CP children and their siblings. This could be attributed to increased parental awareness and supervision for both of their children, irrespective of each child’s manual dexterity.

Dental caries estimation was performed using the first known systematic caries index, the DMFT index. The dft index was a modification of the DMFT index accepted by WHO for primary teeth, representing decayed and filled teeth. The dft index ignores missing teeth to get around the exfoliation problems in the transient dentition. This study included children from 5 to 12 years of age. Children aged 5 to 6 years were in their primary dentition period, and those from 6 to 12 years were in their transient mixed dentition period. The caries indices for mixed dentition and primary dentition were done separately. The present study showed significantly higher dft values in CP children, in accordance with the study of Vandal et al., who found that this could be due to poor neuromuscular coordination, poor oral hygiene practices, and lack of awareness on the part of parents. Another contributory factor is the sweetened medications (carbamazepine most commonly and sometimes even herbal formulations) given to the CP children to control seizures and other medical problems.^8^ The DMFT values in the present study were lower in the study group than in the control group, but the results were not significant. Though not statistically significant, poorer oral hygiene and more frequent dental erosion and dental trauma in CP children were evident, possibly making their teeth more prone to dental caries in the primary dentition stage of growth.^9^,^10^ Poor muscular coordination accompanied with varying degrees of mental retardation is also a possible causative factor for the increased caries occurrence in CP children.

The mean pH of the study group was 4.61, which was slightly lower than the control group (5.68), and this was statistically significant (*P*<0.05). This finding was similar to the studies of Radha et al.^11^ and dos Santos et al.,^12^ demonstrating lowered pH among CP individuals.^12^ The present study showed no significant difference in buffering capacity between the two groups, which is contrary to the study by dos Santos et al., who found a statistically significant difference in buffering capacity in CP children.^12^ However, our study results were in accordance with the results obtained by Subramaniam et al. with regard to buffering capacity.^13^ Our study group showed a mean salivary flow rate of 3.25 mL/min as compared to a slightly higher flow rate of 5.14 mL/min in their healthy siblings. This difference was highly significant (*P*<0.01). The results of the present study contradict those of a similar study by Subramaniam et al. that compared CP children without mental retardation and normal children; Subramaniam et al. found no significantly higher salivary flow rate values, and they attributed this variation to the fact that flow rate and salivary content change with age.^13^

This study established a two-tailed correlation of caries with salivary flow rate (*P*<0.05). It has been shown that individuals with CP have increased drooling, which is problematic for maintaining their daily hygiene needs. Earlier studies have noted that this increased drooling is not due to hypersecretion of saliva, but rather to the compromised oral motor control in this pediatric subgroup.^14^ Adequate hydration of CP individuals is necessary to achieve an unstimulated salivary flow. Maintenance of good hydration in CP individuals is highly challenging; as a result the unstimulated salivary flow rate is also highly variable.^15^ In spite of these factors, this study shows that salivary flow rate can serve as a promising tool for caries risk assessment.

## CONCLUSION

The present study showed lower salivary pH and salivary flow rate, higher dental caries occurrence, and fair oral hygiene in CP children in comparison with their healthy siblings. The following conclusions were drawn from this study:

1. Low pH and low salivary flow rate might serve as risk factors for dental caries in the CP population.
2. Salivary flow rate could be used as a screening tool for assessing subjects at risk for dental caries in the CP population.

## Abbreviations

CP: cerebral palsy
dft: decay/filled teeth–primary dentition
DMFT: decay/missing/filled teeth– permanent dentition
OHI-S: Simplified Oral Hygiene Index
WHO: World Health Organization.
